# Understanding the views of adult migrants around catch-up vaccination for missed routine immunisations to define strategies to improve coverage: A UK in-depth interview study

**DOI:** 10.1016/j.vaccine.2024.04.005

**Published:** 2024-05-10

**Authors:** Anna Deal, Alison F. Crawshaw, Maha Salloum, Sally E. Hayward, Jessica Carter, Felicity Knights, Farah Seedat, Oumnia Bouaddi, Nuria Sanchez-Clemente, Laura Muzinga Lutumba, Lusau Mimi Kitoko, Sarah Nkembi, Caroline Hickey, Sandra Mounier-Jack, Azeem Majeed, Sally Hargreaves

**Affiliations:** a The Migrant Health Research Group, Institute for Infection and Immunity, St George’s, University of London, UK; b Faculty of Public Health and Policy, London School of Hygiene and Tropical Medicine, London, UK; c Hackney Congolese Women Support Group, UK; d Hackney Refugee and Migrant Forum, UK; e Department of Primary Care & Public Health, Imperial College London, UK; f Global Health Institute, University of Antwerp, Belgium

## Abstract

**Background:**

The World Health Organization’s (WHO) Immunization Agenda 2030 emphasises ensuring equitable access to vaccination across the life course. This includes placing an emphasis on migrant populations who may have missed key childhood vaccines, doses, and boosters due to disrupted healthcare systems and the migration process, or differing vaccination schedules in home countries. Guidelines exist in the UK for offering catch-up vaccinations to adolscent and adult migrants with incomplete or uncertain vaccination status (including MMR, Td-IPV, MenACWY, HPV), but emerging evidence suggests awareness and implementation in primary care is poor. It is unclear whether patient-level barriers to uptake of catch-up vaccinations also exist. We explored experiences and views around catch-up vaccination among adult migrants from a range of backgrounds, to define strategies for improving catch-up vaccination policy and practice.

**Methods:**

In-depth semi-structured interviews were carried out in two phases with adult migrant populations (refugees, asylum seekers, undocumented migrants, those with no recourse to public funds) on views and experiences around vaccination, involving a team of peer researchers from specific migrant communities trained through the study. In Phase 1, we conducted remote interviews with migrants resident in the UK for < 10 years, from diverse backgrounds. In Phase 2, we engaged specifically Congolese and Angolan migrants as part of a community-based participatory study. Topic guides were developed iteratively and piloted. Participants were recruited using purposive, opportunistic and snowball sampling methods. Interviews were conducted in English (interpreters offered), Lingala or French and were audio-recorded, transcribed and analysed using a thematic framework approach in NVivo 12.

**Results:**

71 participants (39 in Phase 1, 32 in Phase 2) were interviewed (Mean age 43.6 [SD:12.4] years, 69% female, mean 9.5 [SD:7] years in the UK). Aside from COVID-19 vaccines, most participants reported never having been offered vaccinations or asked about their vaccination history since arriving in the UK as adults. Few participants mentioned being offered specific catch-up vaccines (e.g. MMR/Td-IPV) when attending a healthcare facility on arrival in the UK. Vaccines such as flu vaccines, pregnancy-related or pre-travel vaccination were more commonly mentioned. In general, participants were not aware of adult catch-up vaccination but regarded it positively when it was explained. A few participants expressed concerns about side-effects, risks/inconveniences associated with access (e.g. links to immigration authorities, travel costs), preference for natural remedies, and hesitancy to engage in further vaccination campaigns due to the intensity of COVID-19 vaccination campaigns. Trust was a major factor in vaccination decisions, with distinctions noted within and between groups; some held a healthcare professional’s recommendation in high regard, while others were less trusting towards the healthcare system because of negative experiences of the NHS and past experiences of discrimination, injustice and marginalisation by wider authorities.

**Conclusions:**

The major barrier to adult catch-up vaccination for missed routine immunisations and doses in migrant communities in the UK is the limited opportunities, recommendations or tailored vaccination information presented to migrants by health services. This could be improved with financial incentives for provision of catch-up vaccination in UK primary care, alongside training of healthcare professionals to support catch-up immunisation and raise awareness of existing guidelines. It will also be essential to address root causes of mistrust around vaccination, where it exists among migrants, by working closely with communities to understand their needs and meaningfully involving migrant populations in co-producing tailored information campaigns and culturally relevant interventions to improve coverage.

## 1 Introduction

Adult migrant populations in Europe may have missed key childhood vaccine doses due to disrupted healthcare systems, differing vaccination schedules and availability of vaccines in their home countries, putting them at risk of vaccine-preventable diseases (VPDs) [1], [2]. Migrant groups have previously been involved in VPD outbreaks in Europe, with one of the major factors being low immunisation coverage [1], [3], [4], [5], [6]. Although many adult and adolescent migrants are under-immunised according to their host country’s vaccination schedule, catch-up vaccinations are rarely offered routinely by vaccination programmes in most European countries, including the UK [7], [8].

**Fig. 1:**
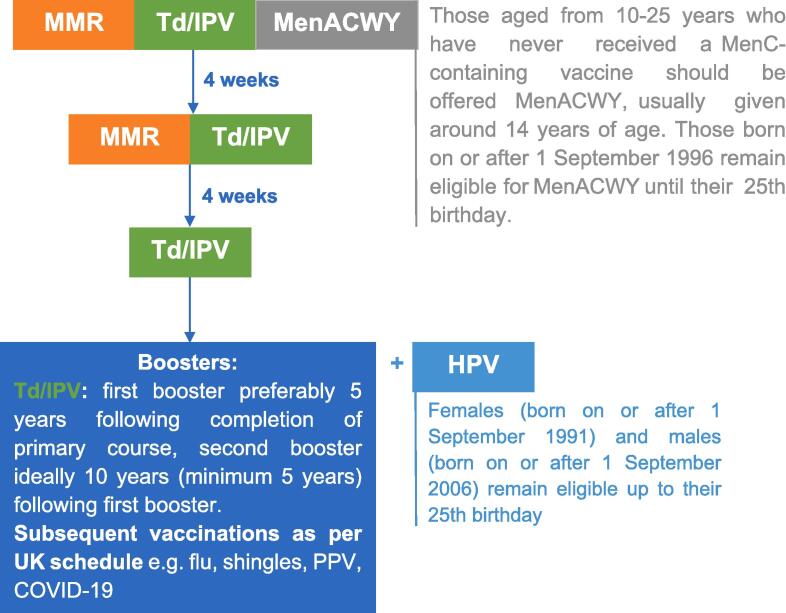
Catch-up vaccination schedule for those over 10 years old with unknown or uncertain immunisation status in the UK, reproduced from [12], [13].

**Fig. 2:**
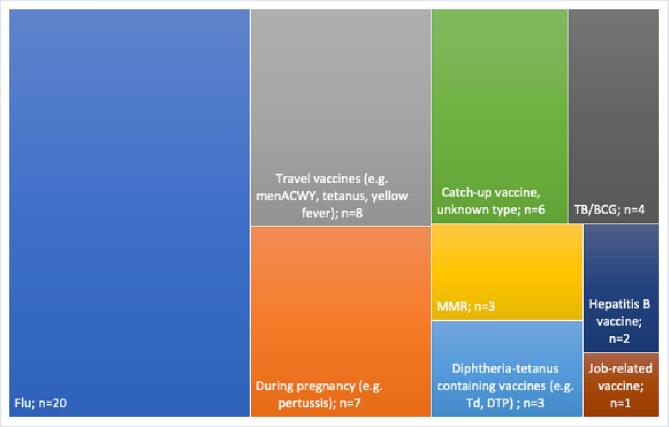
Relative frequencies of participants reporting they had received specific vaccinations since arriving in the UK.

This is despite the World Health Organization’s (WHO) Immunization Agenda 2030, and recent guidelines from the European Centre for Disease Prevention and Control (ECDC), and other reports which emphasise ensuring equitable access to vaccination across the life course, including for under-immunised migrant groups [9], [10], [11]. In the UK, the National Institute for Health and Care Excellence (NICE) has recently produced a set of evidence-based guidelines on increasing vaccination uptake in the general population [12]. These guidelines specifically highlight the importance of identifying populations with low vaccination uptake, including newly arrived migrants and asylum seekers, and ensuring vaccination is offered to all individuals – including adolescents and adults – who are eligible for routine vaccination. Those who arrive in the UK missing doses of key childhood vaccinations or have unknown or uncertain immunisation status are eligible for catch-up doses of vaccinations known or suspected to have been missed. This includes two doses of Measles-Mumps-Rubella (MMR), three doses of Tetanus-diphtheria-inactivated polio virus (Td/IPV), and Meningitidis ACWY (MenACWY) see Fig. 1 [13]. Human papilloma virus (HPV) is also recommended to be offered to under 25s, which is a newer, more expensive vaccine that may not yet be widely available in many low- and middle-income settings (Fig. 2).

However, despite guidelines in place emphasising equitable access to vaccination across the life course, the COVID-19 pandemic highlighted major inequities in access to immunisation services for migrants in the UK and Europe. For example, access was hindered in some migrant groups by a range of unique personal, social and physical barriers. These included difficulty understanding the local healthcare system, language barriers, perceived lack of entitlement to vaccinations, low trust in health systems and/or being unable to afford the indirect costs of attending a vaccination appointment [2], [14], [15], [16], [17]. The 2022 UK NICE guidelines emphasise the need to learn from inequalities identified and initiatives attempted during COVID-19 vaccine roll-outs. This experience should contribute to overcoming barriers in the context of routine vaccination, for example, by considering a flexible approach in terms of locations and opening hours and involving local people in making decisions around accessibility [12]. Low vaccine confidence and vaccine hesitancy are also known to exist in some migrant communities, often rooted in experiences of marginalisation, racism and structural violence in host countries, as well as historical medical injustices suffered by some minority ethnic groups [2], [14], [15], leading to fears around vaccination campaigns using them as ‘guinea-pigs’ [3], [15], [18].

While some evidence exists on migrants’ views around childhood and COVID-19 vaccination, views around adult catch-up vaccination are yet to be fully elucidated, and this is vital to support the development of tailored and targeted catch-up vaccination programmes. We therefore explored experiences and views on barriers to catch-up vaccination among adult migrants in the UK from a range of backgrounds.

## 2 Methods

### 2.1 Study and interview design

This analysis is based on in-depth semi-structured qualitative interviews carried out in two phases, which qualitatively explored views of different migrant populations around routine and catch-up vaccination. Phase 1 specifically aimed to recruit migrants across a various immigration statuses, nationalities, lengths of stay in the UK and religious backgrounds to gain a broad overview of views and experiences around vaccination. The interviews were carried out remotely (either over the phone or through video call) across 17 months (September 2020 to January 2022). The second phase (January-March 2022) consisted of in-person interviews exploring views around catch-up and COVID-19 vaccination with a specific community group (A Congolese group with refugee and migrant members from the Democratic Republic of Congo and Angola), as part of a qualitative community-based participatory research (CBPR) study and was co-designed and conducted in partnership with Hackney Congolese Women Support Group (LML, LMK, SN) and Hackney Refugee and Migrant Forum (CH) [19].

For both phases, topic guides were developed by the research team comprising AD, AFC, SH, SMJ, SEH (academic researchers) and JC, FK (General Practitioners), with specific input from a wider project board comprising migrant representatives from a range of different nationalities and backgrounds. Topic guides were developed through iterative cycles and informed by the situation of the pandemic and the progression of the UK COVID-19 vaccine roll-out. In the second phase, topic guides were developed and piloted with the involvement of members of the target population and community partners (LML, LMK, SN, CH) through the study’s participatory coalition [20]. Across both phases, participants were asked broadly about their experiences of vaccination before, during and after migration to the UK and views around the acceptability, effectiveness, and accessibility of adult catch-up vaccination. Interviews also included questions about COVID-19 vaccination, given the interviews were carried out as the COVID-19 vaccine roll-out was gathering pace in the UK.

### 2.2 Participant recruitment

In Phase 1, migrant participants were recruited using purposive and snowball sampling, with the aim of recruiting participants from a broad range of nationalities, migration statuses, and age groups. Adverts for the study and participant information sheets were circulated to 20 UK-based migrant support groups (mostly based in South London and chosen for their locality around St George’s, University of London) and on social media. Those who expressed an interest in taking part were contacted by telephone and the study was explained to them with interpreters available on request. In Phase 2, Congolese members of the study team known to the local community (through their Congolese charity and support group) recruited migrant participants through word of mouth, adverts for the study, and snowball sampling techniques. Potential participants received translated participant information sheets at least one week ahead of the interviews and had the opportunity to ask questions and decide whether to participate.

### 2.3 Ethics and informed consent

Ethics was granted by St George’s, University of London Research Ethics Committee (REC 2020.0058 and 2021.0128). For both phases, translated participant information sheets were circulated, and written informed consent was obtained from all participants prior to carrying out an interview.

### 2.4 Data collection

During Phase 1, in-depth semi-structured interviews were conducted by telephone (by AD, MS, SEH) and lasted 30–90 min, with a remote interpreter offered to all participants. In Phase 2, in-depth semi-structured interviews were conducted face-to-face in Lingala or French (by LML, LMK, SN) or in English or with the support of an interpreter (by AFC) and lasted 15–50 min. Interviewers were either social scientists by training (AD, MS, SEH, AFC) or peer researchers with lived experience of migration (LML, LMK, SN). The variances this may have produced in terms of power dynamics were taken into account through a process of active reflexivity in the analysis methods. Participants from both phases were compensated with a shopping voucher or gift card, as per INVOLVE NIHR criteria [21] for participant involvement in research studies. Interviews were audio-recorded, translated (if applicable), and transcribed verbatim; all transcripts were checked for accuracy and anonymised. Data collection ended when saturation was reached, and no new concepts were arising.

### 2.5 Analysis

We based our analysis on the thematic framework approach proposed by Srivastava and Thompson, designed to bridge the gap between qualitative and applied policy research [22]. Data familiarisation was done concurrently by two researchers (AD and AFC) and a preliminary framework of topic summaries and codes was designed using a combination of inductive and deductive approaches. Codes were mainly semantic and were refined during analysis, where required. Topic summaries included ‘experiences of vaccination in home country’, ‘experiences of vaccination in the UK’ and ‘barriers and facilitators to catch-up vaccination’ and ‘moral intuitions’. The codes in ‘moral intuitions’ were based on the six automatic intuitions described in the Moral Foundations Theory developed by Graham et al (2011), which influence people's judgements [care (about others' wellbeing), fairness (concerns about proportionality), loyalty (in/out-group relations), authority (following rules/traditions), sanctity (purity and ‘cleanliness’), and liberty (about freedom)] [23], [24]. Data was coded in NVivo by AD and AFC in NVivo 12. While data were collected around views on COVID-19 vaccination, which has been published elsewhere [15], in this analysis we aimed to focus on discussions around experiences of routine and catch-up vaccination, except when points made regarding COVID-19 vaccines were clearly transferable to routine vaccination.

## 3 Results

### 3.1 Participant demographics and characteristics

71 participants were included in this analysis from a diverse range of migrant backgrounds and recruited across two separate studies, see Table 1. Overall, 44 (62 %) of participants identified as refugees or asylum seekers, with the remaining 38 % reporting a range of immigration statuses, such as working visas with no recourse to public funds or naturalised British. The mean age of participants was 43.6 [SD:7.2] years, with the mean age considerably higher in the Phase 2 recruitment cohort than in Phase 1. The average time since arrival in the UK across the two cohorts was 9.5 years (SD:7 years), and 61 % originated from the WHO African region, followed by 21 % from the Eastern Mediterranean region. 58 % of participants reported their religion as Christianity and 28 % as Islam.

**Table 1:** Characteristics of qualitative interview participants (n = 71).

**Characteristic**	**n (%)**	**Phase 1n = 39**	**Phase 2n = 32**
**Migrant status**			
Asylum seeker Refugee Undocumented No recourse to public funds/any other visa British (naturalised) Prefer not to say	25 (35 %) 19 (27 %) 9 (13 %) 7 (10) 6 (8 %) 5 (7 %)	19 (49 %) 6 (15 %) 9 (23 %) 5 (13 %) 0 (0) 0 (0)	6 (19 %) 13 (41 %) 0 (0 %) 2 (6 %) 6 (19 %) 5 (16 %)
**Age in years, mean (SD)**	**43.6 (7.2)**	**36.1 (7.6)**	**52.6 (11.0)**
18–30 31–45 46–60 Over 60	8 (11 %) 36 (51 %) 20 (28 %) 7 (10 %)	8 (21 %) 29 (74 %) 2 (5 %) 0 (0 %)	0 (0 %) 7 (22 %) 18 (56 %) 7 (22 %)
**Gender**			
Female Male	49 (69 %) 22 (31 %)	25 (64 %) 14 (36 %)	24 (75 %) 8 (25 %)
Time since arrival in the UK (years), mean (SD) *	**9.5 (7.0)**	**5.7 (3.2)**	**14.3 (7.5)**
0–5 6–10 11–20 >20 years Not available	22 (31 %) 22 (31 %) 16 (23 %) 7 (10 %) 4 (6 %)	18 (46 %) 18 (46 %) 1 (3 %) 0 (0) 2 (5 %)	4 (13 %) 4 (13 %) 15 (47 %) 7(22 %) 2 (6 %)
**WHO Region of origin**			
African Region (Angola, Democratic Republic of Congo, Mauritius, Nigeria, Uganda, Zimbabwe, other/unknown) Eastern Mediterranean Region (Afghanistan, Egypt, Iraq, Pakistan, Syria, other/unknown) European Region (Albania, Kyrgyzstan, Turkey, Ukraine, other/unknown) South-East Asian & Western Pacific Region (India, Sri Lanka) Region of the Americas (Venezuela) Western Pacific (Malaysia)	43 (61 %) 15 (21 %) 6 (8 %) 5 (7 %) 1 (1 %) 1 (1 %)	11 (28 %) 15 (38 %) 6 (15 %) 5 (13 %) 1 (3 %) 1 (3 %)	32 (100 %) 0 0 0 0 0
**Currently have children < 18 years of age living in household**			
Yes No Information not available	42 (59 %) 23 (32 %) 6 (8 %)	27 (69 %) 6 (15 %) 6 (15 %)	15 (47 %) 17 (53 %) 0 (0 %)
**Religion** Christianity Islam Other/none Information not available	41 (58 %) 20 (28 %) 2 (3 %) 8 (11 %)	9 (23 %) 20 (51 %) 2 (5 %) 8 (21 %)	32 (100 %) 0 0 0

*Where respondents answered the question ‘Time since arrived in the UK’ with “more than 10 years”, this was assigned the value of 10 years in the continuous distribution/mean calculation; “more than 20 years” was assigned the value of 20 years; “more than 25 years” was assigned the value of 25 years.

Table 1: Characteristics of qualitative interview participants (n = 71).

### 3.2 Vaccination history and previous experiences of vaccination

#### 3.2.1 Views and experiences of vaccination in home countries

Participants reported a range of recollections of their childhood vaccination history and had different views on the vaccination systems in their home countries. Most reported being unsure of which vaccinations they had received, and some suggested that they or others in their community may not have been vaccinated due to disruption of their home country’s healthcare system, due to war, instability or lack of funding. Several participants from Phase 1 mentioned that vaccination was not free in their country of origin or that, particularly in rural areas, many vaccines were simply not available when they were growing up and therefore *“the children are not immunised enough”*. By contrast, participants from the Democratic Republic of Congo (DRC) specifically recalled participating in mass immunisation campaigns during their childhood, although few could name the vaccines they had received. Many of these participants mentioned that they had visible scars or keloids from specific vaccines received in childhood, which had made them associate vaccination with fear or pain. Some participants suggested they had low confidence in the healthcare or vaccination system in their home country, as they considered that vaccines may be lower quality, not be stored well or that healthcare staff may not follow safe protocol.

*“When I was back home, they can use one needle to treat many people. I don't know if they stopped it. But here, when they use one needle, you will see them put it in the bin. They don’t repeat it again for any other person”*

*P26, asylum seeker, female*

*“I wouldn’t be really comfortable taking [a vaccine] in [my home country] because I know they wouldn’t really care. They may easily left it out of the fridge”*

*P70, No recourse to public funds, female*

Some participants mentioned that vaccine mandates were enforced in their country of origin, which they usually viewed in a negative light, compared to the UK where some felt more empowered by being offered a choice.

*“In [home country], it’s not like that, they are forcing and it is compulsory. Otherwise, they will ask unnecessary questions and they will blame us, something like that. Some people, if they like or don’t like, they’re forced to do the vaccine. It is not good”*

*P21, asylum seeker, male*

Around half of participants suggested that some form of childhood vaccination records were given in their country of origin (Table 2), but nearly all suggested that these were in paper form and several suggested that their parents would have been unlikely to keep or understand these records.

**Table 2:** Reported frequencies of possessing a vaccination card.

**Given routine/childhood vaccination card in country of origin (n = 54 asked)**	
Yes	23 (43 %)
No	24 (44 %)
Don’t know	7 (13 %)
**Brought their own routine/childhood vaccination card to the UK (n = 28 asked)**	
Yes	8 (29 %)
No	16 (57 %)
Don’t know	4 (14 %)

Table 2: Reported frequencies of possessing a vaccination card.

#### 3.2.2 Experiences of vaccination after arrival in the UK as an adult

Excluding COVID-19 vaccines, most participants reported having never been offered vaccination or asked about their vaccination history by a healthcare professional since arriving in the UK as an adult, despite participants’ mean time since arrival in the UK being 9.5 [SD:7] years. Most participants with young children suggested that their children’s vaccination records had been checked by a GP and catch-up vaccinations offered, but for adolescents and adults this was rarely done.

*“[They only asked] for the little one [child], not even the older one. The big one is 15, so they didn’t ask for anything. When he came here, he was 12, they didn’t ask for anything. But little one, they asked if he had this vaccination”*

*P6, asylum seeker, female*

*“In my country, if you go to a GP they will always ask you for your record of immunisation. I don’t know what if it’s what [they] do here or if it’s because they don’t care. So, we feel that [in the UK] we are not, we haven’t had the opportunity to be screened on these diseases or to receive our immunisations”*

*P60, refugee, female*

The few who mentioned that they had been offered a vaccine by a GP or healthcare worker reported being offered a wide range of vaccines, highlighting inconsistency in terms of what is offered. Participants most commonly reported being offered flu vaccination, followed by travel vaccinations, such as meningitis before going on Hajj or tetanus/yellow fever before traveling to their home country. Some participants reported having received vaccinations in pregnancy (e.g. rubella or pertussis) and a small number recalled being offered some type of catch-up vaccination, often during stays in initial asylum accommodation or during resettlement processes, but most could not remember or had never been sure what they had received.

There was a strong sense that most participants had never considered or been given any information on receiving missed routine vaccinations (excluding flu and Covid-19 vaccination) as an adult whilst in the UK. Many participants said that they would have been interested in receiving them or finding out more, if they had known this was a possibility. Participants were generally more used to discussing vaccination of children. In general, women with children appeared more informed and expressed more views on different vaccinations.

*“I don’t know if there are right now vaccinations that I can take, I’m not sure. And the reason behind that, actually, there is no clear information”*

*P44, refugee, male*

*“I was surprised that you ask me about vaccination when you get into UK. This one surprise me because it is the first time I heard [about it] from you”*

*P12, asylum seeker, male*

### 3.3 Barriers and facilitators to catch-up vaccination

#### 3.3.1 A balancing act between risk and trust: Personal and structural barriers to catch-up vaccination

Participants reported experiencing a range of barriers to vaccination, including systemic barriers (such as language or financial constraints) and personal barriers, which were often deeply rooted in ideas around risk, trust and cultural or religious norms. Concerns around side-effects or harmful consequences of vaccination were often discussed as a perceived risk, particularly among those who appeared to put importance on sanctity or liberty. Several participants suggested that they did not consider vaccines to be effective, and some suggested that following good hygiene practices or allowing their immune system to be naturally challenged offered a better alternative to receiving a vaccine:

*“You can get a jab this year but […] you might still get a flu or every year the flu virus is changing so it’s not that effective. So instead of getting a vaccine you can just make sure that you are safe, clean and hygiene and other things. So that’s what I think”*

*P44, refugee, male*

Some participants also expressed a sense of vaccination “fatigue” or a feeling of being ‘over-vaccinated’ after COVID-19 vaccination campaigns, which made them reluctant to consider receiving catch-up vaccinations:

*“I’m not really sure [about getting a catch-up vaccine] because there’ve been so much vaccination nowadays. So, we can’t be taking this and that”*

*P71, migration status not reported, female*

Our study participants expressed different levels of trust towards healthcare professionals, health systems and other authorities, and this was in part linked to formative experiences with these systems. Participants who had experienced insensitivity from a healthcare worker said they would be less likely to accept offered vaccinations. First experiences in the UK after arrival appeared to have a particularly strong impact on trust and subsequent likelihood to accept vaccination. Fears around data sharing between healthcare services and immigration authorities were also brought up in relation to trust, particularly those with undocumented status. These negative experiences contributed to participants perceiving a vaccination decision as high risk, and something they would be less likely to engage with. For example:

*“A lot of people are afraid, that’s the thing. Once you don't have that document, so you tend to be afraid to do some certain things. I know some of my, maybe three, friends that have passed away because they are afraid to go to the hospital. They're afraid to maybe they're going to ask them some document and all that” P26, asylum seeker, female*

Some participants with children, particularly mothers, associated being able to look after and protect their child(ren) and family as the most important safety-related aspect when making a vaccination decision for themselves. This led some to suggest that they would reject a vaccination if they felt it would cause them harm that would impact their ability to care for their family, or to accept vaccination if the risk of contracting the VPD was perceived to be higher than the risk of harm from the vaccine.

#### 3.3.2 Information as a facilitator: The importance of having access to a trusted messenger or a relatable figure

We noticed some clear patterns in our data suggesting that certain groups and individuals appear to lean towards authority as a ‘moral intuition’ (as described in the Moral Foundations Theory developed by Graham et al (2011)), putting higher trust in authority figures when making a vaccination decision. For example, several participants stated they would make a vaccination decision solely on the recommendation of a specific authority figure (usually a GP, religious leader or official/government recommendation), which was suggested as being rooted in cultural norms, in some cases. For example, many participants from certain South Asian communities showed high trust in a doctor’s recommendation:

*“The [GP] told, this [vaccine] is good for you, and there was no explanation given, but when they said, this is good for you, I agreed […] when you think about it, generally, doctors’ word is gospel back home. When a doctor says, yes, this is good for you, nine out of ten people do not ask any questions, you know what I mean?”*

*P14, asylum seeker, male*

For those whose moral foundations centred more on liberty, sanctity or care, rather than authority, having information clearly explained by a healthcare professional was an important factor influencing many participants’ decisions to receive vaccination. When the reasons for them needing a vaccine had been well explained, participants who had been offered catch-up or routine vaccination [excluding Covid-19 vaccines] almost always stated they had accepted. It is possible that these explanations effectively conveyed the risk of remaining unvaccinated or reassurance around the safety of vaccination in a way that resonated with the individual. For example:

***“****They were asking me if I had this vaccination and I said, no, because I remembered. They said, okay, we have to do it. It doesn’t affect you if you have done it before, just to make sure. I said, okay. I accept them. They’re done”*

*P18, refugee, female*

In contrast, one participant explained that when migrants were not provided with information in a language or way that they could understand, they were likely to refuse vaccination, indicating a clinical shortcoming and lack of cultural sensitivity:

*“When I was pregnant, I saw many other pregnancies from other backgrounds and they cannot talk English or they are recently arrived to UK. So, I saw that when the midwife or NHS offered them vaccine, they said, no, thank you. I asked them what’s this vaccine they refuse, and they don’t know what is it. They said no but they don’t know”*

*P3, refugee, female*

While for some individuals and communities, receiving information or a recommendation from an authority figure or healthcare professional was a major factor in vaccine decision making, some participants appeared to relate more to other known or trusted figures, such as friends, family or community members. These participants discussed how stories or information from those around them influence their decision making around vaccination, particularly when they were hesitant or evaluating whether to get vaccinated. Regarding COVID-19 vaccination, one participant said:

*“I actually I have a friend […] and nothing happened to him [after being vaccinated], and he’s black too, he’s black, nothing happened to him”*

*P32, asylum seeker, female*

Another participant said,

*“Yes, because I saw my neighbour. He got it, he have no problem. No bad reaction. Nothing. And then, that’s why I went.”*

*P53, indefinite leave to remain, male*

Many participants, particularly those originating from sub-Saharan African countries, tended to value liberty, autonomy and being able to make their own informed decisions. Some said that they would undertake their own research about a vaccination before making a decision, so that they could feel happy about their choice.

*“[If] I don’t know what I’m taking this for, I won’t take it. […] You can’t just take vaccine without looking at… It’s like taking a drug, you don’t know why you are drinking medicine. You have to read the label, see how it works […] Me, I have to go to the internet and read it and see which work it does before I can [take a vaccine]”*

*P15, undocumented, female*

#### 3.3.3 Vaccination-related communication strategies: reminders and mandates – barrier or facilitator?

Participants had contrasting views around vaccination reminders and mandates. Some participants, who generally leaned towards authority and care as moral intuitions, suggested that regardless of their personal motivations or views on vaccination, they had accepted specific vaccines after being sent repeated reminders or letters inviting them to be immunised.

*“I was very worried to take [the vaccine] and so I refused […] But because they keep sending texts and they keep sending post to the home […] so I start to change my mind. I call them and they sent a post to me to the home, saying if you do not manage with English, there is many, many numbers to call, the language you want […] So, I start to change my mind […] after two days and I got it”*

*P3, refugee, female*

By contrast, many others (particularly the Congolese participants) said that they found COVID-19 vaccination reminders and mandates to be coercive and controlling, which was off-putting, and led them to question if there were more sinister motives behind the COVID-19 vaccination programme. They also compared their experiences of reminders with the flu vaccination, which they said they had never been “forced” to receive.

*“I have been constantly receiving letter pushing me to receive vaccine. […] I would do it voluntarily but not by force. Now they are forcing people and I don’t know what is hidden behind this vaccine?”*

*P66, asylum seeker, male*

## 4 Discussion

The importance of developing services to deliver vaccination across the life course is widely accepted, with a renewed focus now on driving up coverage for routine immunisations in under-immunised adults and adolescent groups, such as migrants, who may have missed key vaccines and doses in childhood. Our study found that in the UK, most migrants have never had their vaccination history checked, even after an average of 9.5 [SD:7] years in the country and are not being offered catch-up vaccinations when they present to health services, even as part of a new patient health check. Participants mostly reported having been offered seasonal vaccines (such as influenza) or travel vaccines (e.g. MenACWY before Hajj, yellow fever) rather than catch-up routine vaccinations such as MMR, Td/IPV or HPV), suggesting that catch-up vaccination is not currently prioritised in primary care and that healthcare professionals may not be aware of catch-up guidelines or have enough incentive, training or time to follow them. This is a finding which was also reflected in our 2022 UK qualitative study with healthcare professionals [16]. Participants in our study reported facing a range of personal and structural barriers to accessing vaccination in the UK. Trust – either in vaccination, the healthcare system or wider authorities – was often reported as a key factor influencing vaccination decisions in the UK. The importance of having a trusted, accessible, and relatable source of information was highlighted, with nearly all participants suggesting they had never been given any health information around catch-up vaccination since arriving in the UK.

While the UK has specific guidelines, for example, for the vaccination of those with uncertain or incomplete immunisation status, which prioritises MMR, Td-IPV and MenACWY catch-up vaccination [13], our findings fit with existing evidence suggesting that implementation of these guidelines is currently poor [16]. A recent qualitative study with primary care professionals in the UK showed that delivery models are often diverse and fragmented, where they exist, with major inhibitors to implementation including a lack of training and knowledge of guidance among staff; unclear or incomplete vaccine records; and lack of incentivisation (including financial) and dedicated time and care pathways [16]. A Europe-wide survey also found that while most countries have some form of guidelines on catch-up vaccination, few specifically mention migrants, and the majority reported that guidelines were either incompletely applied (16/32) or never applied in practice (2/32) [7]. Data from a recent needs assessment in Southwark, London, show that catch-up vaccination does happen as part of initial health checks for asylum seekers in UK Home Office-provided accommodation [25], but this may vary highly between local authorities. There is little national data around implementation of catch-up vaccination in these specific settings. The UK’s NICE guidelines for increasing vaccination uptake in the general population currently put an emphasis on ensuring that all healthcare staff receive training on who is eligible for vaccination and promote opportunistically identifying people who may be under-immunised, such as registration at a primary care practice, in community health clinics or at pharmacies [12]. However, this study, along with others [16] suggest that this does not happen in practice, despite the majority of participants suggesting they may be willing to receive catch-up vaccination if they were recommended it. However, opportunistically identifying those eligible for catch-up vaccination does risk missing or excluding those who may have limited interactions with mainstream health services, such as undocumented migrants. The ECDC has published guidance on catch-up vaccination in children and adult migrants on arrival, designed to support EU/EEA Member States to develop national strategies, also emphasises the importance of identifying the key interactions along the migration trajectory for vaccination records to be checked and vaccinations offered [10].

Another key finding from this study was the importance of both trust and information as both a barrier and facilitator of vaccination uptake in migrant populations, which reflects findings from existing literature investigating vaccine confidence among migrant and ethnically minoritized groups[14], [15], [26], [27], [28]. We found that participants reported a range of views, expectations and experiences around trust, with previous experiences in the UK, particularly those soon after arrival, appearing to have a particularly strong impact on trust and subsequent likelihood to accept vaccination. Participants expressed different views around information, communication strategies and how they related this to a sense of trust or confidence in vaccination. Patterns in the data around expectations of communication and trust among different cultures or communities included that those from a South Asian background appeared to put a high level of trust in a GP’s recommendation, and often reported not wanting additional information, whereas those from a Black African background were more likely to report wanting information on the vaccination they would be receiving before making a decision. However, using the moral foundations theory as a framework also allowed us to identify the deeply personal drivers of trust and vaccine confidence, a phenomenon that has previously been described in terms of moral intuitions by Schmidtke et al [24], who found in a survey study on COVID-19 vaccination that four specific moral intuitions (liberty, authority, care and sanctity) were associated with vaccine hesitancy [24]. Our study found that those who put importance on moral intuitions such as liberty and sanctity were more likely to put importance on having access to sufficient information before vaccinating, whereas those who leaned towards authority as a moral intuition were more likely to trust a recommendation of someone they trust. Better understanding the reasons why various sub-populations of migrants align with specific moral intuitions should be an area of future research. This study was not geared towards understanding this phenomenon in depth and we recommend future studies explore up to what point individual moral intuitions, personal experiences and cultural norms and expectations influence vaccine confidence. Across the spectrum of views around trust and expectations around information, having a relatable and trusted messenger was a key facilitator to vaccination. This correlates with previous studies that show the importance of trusted messengers, for example, a study in Cox’s bazaar, Bangladesh that highlighted the importance of local faith leaders as sources of information about vaccination for Rohingya refugees [29], and the success of a local radio host in combatting COVID-19 misinformation in a refugee camp in Kenya [30], [31].

Importantly, we found that hesitant views about specific vaccines (such as against COVID-19 vaccines) or vaccination generally do not always correlate with refusal of vaccination, aligning with finding in past literature [32]. Several participants listed concerns about either a specific vaccine (often COVID-19) or vaccination generally, but also suggested they had later received vaccination, or would still accept vaccination if certain conditions were met (such as receiving specific information). For example, some who suggested they had received a sensitive explanation from a healthcare worker described then accepting a vaccination, whereas a few decided to receive a vaccination after being ‘nudged’ or sent repeated reminders. However, it was also clear that ‘one size does not fit all’, with some participants suggesting they found reminders or texts coercive and made them less likely to accept vaccination. This highlights the importance of understanding communication preferences among different communities or groups and tailoring communication appropriately for the target group, which has been recommended by a range of studies and guidelines [2], [12], [14], [15], [24], but is often still not done well in practice. It was also clear from our findings that many participants had positive views around catch-up vaccination, though they had never received one due to not knowing that they were available, needed or that they might be eligible for one. Therefore, efforts to increase vaccine confidence are unlikely to translate into increased uptake unless a consistent, proactive system is in place to identify eligible individuals (check vaccination history) and offer vaccination to those who need it. Multi-level approaches are needed to increase uptake of catch-up vaccines across a spectrum of different views about vaccination; addressing system-level barriers as well as individual drivers/determinants; appropriately documenting vaccinations which are given to this mobile population on arrival to the UK; clear recommendations of vaccination from a healthcare worker, and tailored campaigns offering culturally sensitive explanations of why a specific vaccine is being offered.

Box 1 **Recommendations for strengthening delivery of catch-up vaccinations to migrants.**

• Explore and evaluate novel pathways, settings, approaches and funding mechanisms to support the delivery of catch-up vaccination, both within and outside of primary care
• Set and use immunisation targets and financial incentives in primary care
• Facilitate and champion good practice around offering vaccination, including cultural competency, access to interpreters, migrant-inclusive and sensitive practice.
• Provide training to HCPs and raise awareness of the need to check migrants’ vaccination history and opportunistically offer catch-up of key routine vaccinations, including to adolescents and adults
• Tailor vaccination and general healthcare services, involving migrant communities in co-design and decision-making
• Designate adequate funding and infrastructure in primary care to support the delivery of catch-up vaccinations to migrants across the life course and outside of standard age groups for routine childhood immunisations
• Design systems to appropriately document vaccinations which are given to this mobile population on arrival to the UK, to avoid unnecessary repetition of vaccines, which may erode confidence
• Engage relevant stakeholders such as local authorities, NGOs, or the migrant communities themselves in the vaccine delivery process.

While this study has generated new evidence on views and experiences around adult catch-up vaccination, drawing on experiences of participants from a wide range of backgrounds, it has a number of limitations. These include a lack of geographical representation from across the UK (most participants were resident in London or the North East) and lack of representation of some nationalities and age groups. The timing of the studies, which were both done in the context of the COVID-19 pandemic and ongoing COVID-19 vaccination campaigns, may have influenced participants’ views of vaccination in general. However, whilst it is important to acknowledge the context in which these results were situated, this could also be considered a strength of the research, as understanding the influence of COVID-19 vaccine campaigns on confidence in routine vaccination will be key moving forwards, with more research urgently required on this topic. It is also important to note that we did not use any survey tools to formally assess participants’ moral intuitions during this study but used the principle to frame our analysis by observing the frequency of moral intuition codes across individual transcripts.

In conclusion, we have shown that there are a range of important personal and structural barriers to uptake of catch-up vaccination among migrants in the UK. In order to increase catch-up vaccination uptake, policy needs to be shifted towards the prioritisation and financial incentivisation of the provision of catch-up vaccination in primary care, along with training and supporting healthcare professionals to deliver catch-up immunisation as per existing guidelines. Changes in policy should be accompanied by clear, culturally appropriate information campaigns for those likely to be eligible for vaccination, and innovative, collaborative approaches to working with communities to identify and address concerns or low vaccine confidence. These findings are also relevant to implementing catch-up vaccination campaigns for migrant and other marginalised groups in other European and high-income countries and globally, with strategies and recommendations for catch-up vaccination highlighted in a recent WHO report on strengthening immunisation globally in migrant populations [11].

## CRediT authorship contribution statement

**Anna Deal:** Conceptualization, Formal analysis, Investigation, Methodology, Writing – original draft, Writing – review & editing. **Alison F. Crawshaw:** Conceptualization, Formal analysis, Investigation, Methodology, Writing – original draft, Writing – review & editing. **Maha Salloum:** Investigation, Writing – review & editing. **Sally E. Hayward:** Investigation, Writing – review & editing. **Jessica Carter:** Writing – review & editing. **Felicity Knights:** Writing – review & editing. **Farah Seedat:** Writing – review & editing. **Oumnia Bouaddi:** Writing – review & editing. **Nuria Sanchez-Clemente:** Writing – review & editing. **Laura Muzinga Lutumba:** Investigation, Writing – review & editing. **Lusau Mimi Kitoko:** Investigation, Writing – review & editing. **Sarah Nkembi:** Investigation, Writing – review & editing. **Caroline Hickey:** Project administration. **Sandra Mounier-Jack:** Writing – review & editing, Supervision. **Azeem Majeed:** Writing – review & editing. **Sally Hargreaves:** Conceptualization, Investigation, Writing – review & editing.

## Declaration of competing interest

The authors declare the following financial interests/personal relationships which may be considered as potential competing interests: Sally Hargreaves, Alison Crawshaw reports financial support was provided by National Institute of Health Research (NIHR). Anna Deal, Sally Hayward reports financial support was provided by UKRI Medical Research Council. Sally Hargreaves reports financial support was provided by Academy of Medical Sciences. Sandra Mounier-Jack reports financial support was provided by National Institute for Health Research Health Protection Research Unit in Vaccines and Immunisation. Azeem Majeed reports financial support was provided by NIHR Applied Research Collaboration NW London. Azeem Majeed reports financial support was provided by NIHR Biomedical Research Centre. Oumnia Bouaddi reports financial support was provided by La Caixa.

## Data Availability

Data will be made available on request.
